# Self-assessment of eligibility for early medical abortion using m-Health to calculate gestational age in Cape Town, South Africa: a feasibility pilot study

**DOI:** 10.1186/s12978-016-0160-x

**Published:** 2016-04-16

**Authors:** Mariette Momberg, Jane Harries, Deborah Constant

**Affiliations:** Women’s Health Research Unit, School of Public Health and Family Medicine, Faculty of Health Sciences, University of Cape Town, Anzio Road. Observatory, 7925 Cape Town, South Africa

**Keywords:** Abortion, Medical abortion, Self-assessment, Pregnancy dating, Menstruation, mHealth, Gestational age calculator, South Africa

## Abstract

**Background:**

Although abortion is legally available in South Africa, barriers to access exist. Early medical abortion is available to women with a gestational age up to 63 days and timely access is essential. This study aimed to determine women’s acceptability and ability to self-assess eligibility for early medical abortion using an online gestational age calculator. Women’s acceptability, views and preferences of using mobile technology for gestational age (GA) determination were explored. No previous studies to ascertain the accuracy of online self-administered calculators in a non-clinical setting have been conducted.

**Methods:**

A convenience sample of abortion seekers were recruited from two health care clinics in Cape Town, South Africa in 2014. Seventy-eight women were enrolled and tasked with completing an online self-assessment by entering the first day of their last menstrual period (LMP) onto a website which calculated their GA. A short survey explored the feasibility and acceptability of employing m-Health technology in abortion services. Self-calculated GA was compared with ultrasound gestational age obtained from clinical records.

**Results:**

Participant mean age was 28 (SD 6.8), 41 % (32/78) had completed high school and 73 % (57/78) reported owning a smart/feature phone. Internet searches for abortion information prior to clinic visit were undertaken by 19/78 (24 %) women. Most participants found the online GA calculator easy to use (91 %; 71/78); thought the calculation was accurate (86 %; 67/78) and that it would be helpful when considering an abortion (94 %; 73/78). Eighty-three percent (65/78) reported regular periods and recalled their LMP (71 %; 55/78). On average women overestimated GA by 0.5 days (SD 14.5) and first sought an abortion 10 days (SD 14.3) after pregnancy confirmation.

**Conclusions:**

Timely access to information is an essential component of effective abortion services. Advances in the availability of mobile technology represent an opportunity to provide accurate and safe abortion information and services. Our findings indicate that an online GA calculator would be accurate and helpful. GA could be calculated based on LMP recall within an error of 0.5 days, which is not considered clinically significant. An online GA calculator could potentially act as an enabler for women to access safe abortion services sooner.

## Background

Of the 210 million pregnancies that occur across the globe yearly, nearly one in five women decide to terminate the pregnancy [1]. Globally some 22 million pregnancies are terminated unsafely and nearly all (98 %) of these take place in developing countries [2]. Moreover, 13 % of all maternal deaths continue to be the result of unsafe abortions [3]. Despite induced abortion being legally available in South Africa after a change in legislation in 1996, barriers to accessing safe abortion services continue to exist. The South African Choice on Termination of Pregnancy Act (CTOP) of 1996 promotes a woman’s reproductive right to have an early, safe and legal abortion. As a direct result of this legislation, abortion related mortality has decreased by 91 % [4, 5]. However, despite this legislation there are still major barriers to women accessing abortion services.

Medical Abortion (MA) in the first trimester (up to 63 days gestation) was approved by the South African Medicines Control Council in 2001 and has been provided in non-governmental organization (NGO) clinics and private sector since 2002 and more recently in 2011 in the public health sector in the Western Cape. MA has proved safe, effective and acceptable in both developed and developing countries [6–8]. Despite liberal abortion legislation and considerable strides in terms of providing access to safe abortion services, evidence suggest that many women still opt for illegally performed (backstreet/unsafe) abortions [3, 4, 9]. Reported reasons include: insufficient knowledge about abortion services; perceived poor quality of care; poor knowledge about different abortion methods and negative attitudes of health care providers [4]. The South African Medical Research Council estimated that 36 % of abortions undergone by adolescents aged 13 and 19 in 2008 took place outside a hospital or clinic and were therefore likely to be unsafe [10]. A recent study found 17.5 % of women accessing second trimester abortions said they had attempted to end their current pregnancy prior to coming to the clinic [11]. A significant number (20–25 %) of women request abortion services in their second trimester – whilst the availability of second trimester services is an important aspect of reproductive health care, reducing the prevalence second trimester abortions has several advantages including decreased risk of procedure-related complications and decreased costs to health services [12]. Decision making processes and delays in seeking abortion services are extremely complex, reasons include: indecisiveness to terminate an unwanted pregnancy; irregular periods and poor recall of menses; health service related barriers such as long waiting-periods, shortage of providers and their reluctance to provide such services [12]. A need therefore exists to improving access to safe abortion services whilst simultaneously focussing on improving knowledge of the availability of such services and ways to enhance or facilitate women’s earlier access through the early identifying of a pregnancy.

Mobile health, frequently referred to as m-health, has been defined by the Global Observatory for e-health of the World Health Organization (WHO) as “medical and public health practice supported by mobile devices, such as mobile phones, patient monitoring devices, personal digital assistants and other wireless devices” [13]. mHealth solutions offer opportunities for quick dissemination of information whilst assuring a certain level of confidentiality [14]. Such solutions therefore have the potential to give women greater sexual reproductive health decision making autonomy [15]. Furthermore, to our knowledge investigations employing mHealth for self-assessment in MA has not been explored.

Recent studies employing mobile phone text messages to strengthen sexual and reproductive health services in low-and-middle-income countries has yielded some promising results, providing both mobile coverage and usage are high [16–19]. South Africa has exceptionally high reported mobile phone ownership. In 2012, International Telecommunications Union (ITU) [20] statistics indicated that there were 134.8 pre- and post-paid mobile subscriptions for every 100 people. These figures clearly support the implementation of m-Health projects in the South African context.

In low-resource settings and developing countries where limited information or technical knowledge is available, last menstrual period (LMP) is often used to determine gestational age [21, 22]. A recent systematic review supported the use of LMP to assess gestational age among first trimester abortion seekers [23]. Previous research found women who intended to carry pregnancy to term and knew their LMP date are more likely to overestimate their gestational age [24–26]. In contrast, LMP studies among women who were seeking abortions have shown varied results, with more reporting underestimation of GA [23, 27, 28]. Although the accuracy of mobile and electronic GA calculators have proven more accurate than manual calculators in a clinical setting [29], no studies have been conducted to determine the accuracy of mobile electronic gestational age calculators in a self-administered setting. Moreover, to our knowledge no online GA calculators exist for women who are considering an abortion. The purpose of this research study was to determine women’s acceptability and ability to assess their own eligibility for an early MA (63 days). We hypothesised that women would be able to self-assess their gestational age and thus their eligibility for MA using an online gestational age calculator combined with an algorithm consisting of five screening questions to determine MA eligibility.

## Methods

A convenience sample of abortion seekers were recruited from October to December 2014, from two clinics (a sexual and reproductive health NGO and a government facility) providing early MA in Cape Town, South Africa. The NGO offers both daily first and second trimester abortion services, whereas the government clinic only offers first trimester abortions on Wednesdays. Abortion services at both sites were provided by trained nurse practitioners.

All women seeking an abortion at the clinics on the study recruitment days (Monday and Wednesday) were approached and offered participation in the study. All women who attended the public healthcare facility had an ultrasound performed at a referring hospital prior to being recruited into the study and therefore knew their GA. Eligibility criteria were: being pregnant; willingness to consent to participate; being 18 years of age or older; able to speak and understand English and having a working mobile phone. The participant’s phone did not require Internet access and did not need to be a smart phone. Written informed consent was obtained and confidentiality and anonymity was assured.

**Fig. 1:**
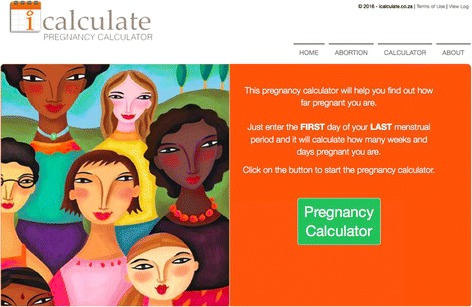
Icalculate mobi-site Homepage

**Fig. 2:**
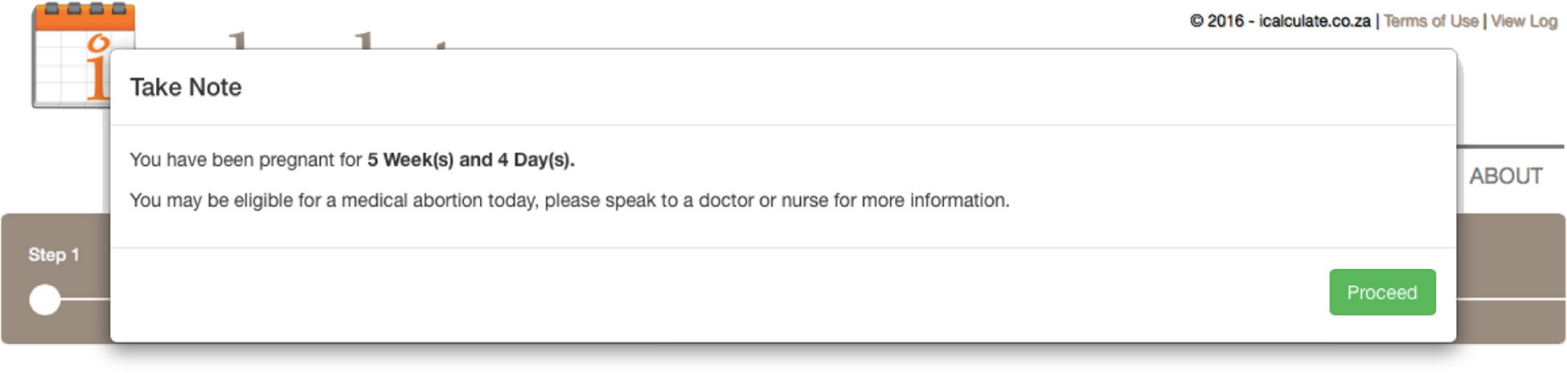
Confirmation message of self-calculated GA

**Fig. 3:**
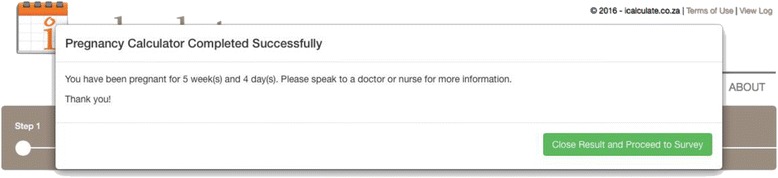
Message to participants who might not be eligible for MA

A website optimised to be viewed and navigated on smaller screens such as mobile phones and electronic tablets (mobi-site) was developed. Participating women were briefly introduced to the website, given instructions on how to use the electronic tablet and asked to follow the on-screen instructions to perform a self-assessment of GA. Participants were then asked to access the mobi-site (www.icalculate.co.za) (Fig. 1) on an electronic tablet supplied by the research assistant. The mobi-site guided participants through the process of determining their LMP and asked five standardised MA-eligibility questions. If LMP was known, the participant had to select the first day of their last menstrual period on the online calendar. If LMP was not known, a calendar with prompts appeared. These prompts asked participants to recall special occasions, public and school holidays to help them recall their LMP. The online calendar was specifically developed for a South African context and displayed school and public holidays that occurred in a given calendar month. If after reviewing the prompts a participant was still unable to recall her LMP date, she was asked to enter an approximate date. Once self-estimated gestation age had been calculated, a message confirming GA and potential eligibility for MA would appear (Fig. 2). The website then routed participants through five eligibility for MA questions: whether they suffer from any bleeding disorders; were taking anti- coagulants; ever had an allergic reaction to MA medication; had pain or bleeding during the pregnancy and whether they had an IUD in situ. If the participant was not eligible for MA based on eligibility questions and (or) GA, a message would appear stating that they that they would need to speak to a health care professional (Fig. 3).

The research assistant sat with the participants whilst they completed the self-assessment and was able to answer any questions they might have. A predetermined form was completed by the research assistant on the number of questions and type of questions asked; whether LMP was known before or after online calendar prompts and the participant’s familiarity with technology. In addition a brief survey was administered by the research assistant using the electronic tablet to explore the following key issues: performing a self-assessment for MA using a website; likelihood to act on the results; perceived accuracy and helpfulness and their current levels of knowledge surrounding abortion services. The survey also collected socio-demographic data including age, educational level, employment status, mobile phone ownership; mobile phone usage and reproductive questions related to contraceptive use and prior abortions. Clinical records were reviewed for confirmed ultrasound GA. Upon completion of the survey; participating women were reimbursed ZAR 50.00 for any expenses incurred.

Ethical approval to conduct the study was obtained from the Human Research Ethics Committee, University of Cape Town and permission was obtained from the participating clinics.

Data were analysed using Stata statistical software (v13, College Station, TX: StataCorp LP). We summarised data on participant characteristics; self-calculated GA; Ultrasound GA; acceptability of using mHealth to self-calculate GA; mobile phone ownership and usage using proportions for categorical variables and means and standard deviations for continuous variables. 95 % confidence intervals (CI) were calculated for proportions, and proportions for categorical variables were compared using chi-square tests or Fisher’s exact tests when appropriate. Missing values were excluded from the analysis, and valid percentages are reported for all results.

## Results

### Socio-demographic characteristics

Seventy-eight women were enrolled and asked to complete a self-assessment on an electronic tablet by entering the first day of their last menstrual period) onto a website which calculated their gestational age. A total of 86 women were approached, of whom one declined and six were ineligible (five were younger than 18 years of age, and one did not own a working mobile phone). One participant survey was incomplete and therefore excluded.

Recruitment took place from two local facilities providing early MA, 46/78 (59 %) women were recruited from a private NGO clinic and 32/78 (41 %) from a public healthcare facility (Table 2). Participant mean age was 28 years (SD 6.8, range 18–42). The majority of participants had completed at least high school education 48/72 62 %. Forty-nine percent (38/78) were employed, 37 % (29/78) were unemployed and 14 % (11/78) were students (Table 1).

**Table 1:** Sociodemographic Characteristics

	Mean	SD	Range
Age	27,51	6,47	18–42
Recruitment Sites (*n* = 78)	%	*n*	
Public Healthcare Facility	41 %	32	
Local (Private) NGO	59 %	46	
Education (*n* = 78)	%	*n*	
Grade 6–11	38 %	30	
Grade 12 +	62 %	48	
Employment (*n* = 78)	%	*n*	
Unemployed	37 %	29	
Employed	49 %	38	
Student	14 %	11	
Reported Phone Type (*n* = 78)	%	*n*	
Basic phone	13 %	10	
Feature/Smart phone	73 %	57	
Did not know	14 %	11	
Reproductive History	Mean	SD	Range
Gravidity (*n* = 78)	2,15	1,06	1–5
Number TOP including current	1,16	0,38	1–2
	%	*n*	
Currently using Contraception^a^	41 %	32	
IUD	3 %	1	
Injectables	23 %	8	
Oral contraceptives	43 %	15	
Male condoms	26 %	9	
Emergency contraception	3 %	1	
Female condoms	3 %	1	
Other	3 %	1	

^a^Dual protection reported by some

### Mobile phone ownership and usage

Seventy-three percent (57/78) of participants reported owning a smart or/feature phone, 13 % (10/78) owned a basic phone and 14 % (11/78) did not know what type of phone they owned. However, most (90 %, 70/78) reported that they were able to access the internet from their phones thus suggesting that smart and feature phone ownership was much higher than reported.

All (78) women used their phones to make and receive calls and to send and receive SMSs. Cross-platform mobile instant messaging chat system applications such as Whatsapp were used by 85 % (66/78), whilst 9 % (6/78) reported using Mxit. The majority of women, 80 % (62/78) used their phones to access the internet and 24 % (19/78) reported doing internet searches for abortion information prior to coming to the clinic.

### Reproductive history

Forty-one percent (32/78) of women participating in our study reported using contraception at the time of enrolment. Oral contraceptive use was most commonly reported failed method of contraception (43 %; 32/78), followed by male condoms (26 %, 9/32) and injectables (23 %, 8/32). One participant reported having an IUD in situ at the time of the survey. Seventeen percent of participants (13/78) reported having a prior abortion. Most participants (91 %, 71/78) had done a pregnancy test prior to presenting at the clinic, of which 65 %, (46/71) had done the pregnancy test at home.

### Online gestational Age calculator

Most participants (91 %; 71/78) found the online GA calculator easy to use and (86 %; 67/78) thought the calculation was accurate and that it would be helpful when considering an abortion (94 %; 73/78). Eighty-three percent (65/78) reported regular periods, most (97 %, 76/78) recalled their LMP month whilst 71 % (55/78) recalled their LMP date. Twenty-nine percent of women (23/78) did not recall their LMP, of whom 43 % (10/23) recalled their LMP after viewing the event calendar prompts and 57 % (13/23) remained unsure and estimated their LMP after reviewing the calendar.

**Fig. 4:**
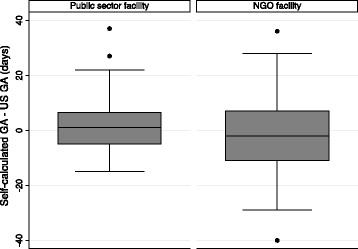
Gestational age difference (GA) Calculation by Site

All women who attended the public healthcare facility had an ultrasound performed at a referring hospital prior to being recruited into the study and therefore knew their GA. However, mean self-calculated GA differences by site were not statistically significant. The median GA by ultrasound at the NGO site was 76 days (IQR, 53–112), compared to 50 days at the public health care facility (IQR, 44–58)(See Table 2 and Fig. 4). Large variances in self-calculated GA when compared with ultrasound GA were observed and variances were significantly greater at the NGO facility (variance ratio test: *f* = 0.460, 2-sided *p* = 0.0251). On average women overestimated GA by 0.5 days (SD 14.5) and first sought an abortion 10 days (SD 14.3) after pregnancy confirmation. In this small sample, 4 % (3/78) self-assessed as eligible for MA (<=63 days), but had U/S GA of >63 days.

**Table 2:** Gestational age (GA) difference in days (Self-calculated GA - ultrasound GA)

	Mean	SD	95 % CI
Public health facility (*n* = 46)	−0,98	16,46	−5.88, 3.90
NGO facility (*n* = 32)	2,63	11,15^a^	−1.39, 6.66
All subject total (*n* = 78)	0,50	14,54	−2.78, 3.78

^a^Variance significantly greater at NGO facility. Variance ratio test: *f* = 0.460, 2-sided *p* = 0.0251

Research Assistant observational notes indicated that the majority of women were familiar with technology and required minimal assistance to navigate both the mobi-site and the MA eligibility screening questions. Only 3/78 (4 %) of women required assistance with the question whether they had an IUD in situ. One woman answered that she did not have an IUD in situ during the self-administered eligibility screening component, yet answered “yes” to the same question when asked by the research assistant during the survey component.

### Abortion legislative knowledge

Participating women were asked whether they knew the legal cut-off period for having a MA and 37 % (29/78) said they did. However, of these only 17 % (5/29) gave the correct answer of 9 weeks. The remainder answered 12 to 19 weeks (59 %, 17/29) and 24 % (7/29) answered 20 weeks.

## Discussion

This feasibility pilot study set out to test women’s acceptability and ability to assess their eligibility for an early MA. Although a number of gestational age calculating websites and downloadable apps exist, none specifically cater for women who are considering terminating a pregnancy. Mobile and electronic gestational age calculators have proven more accurate than manual calculators in clinical settings [29], however studies to ascertain the accuracy of such calculators in a self-administered setting are lacking.

Our findings suggest that women would be able to self-assess their gestational age as well as their eligibility for MA using an online gestational age calculator. The vast majority of participants found the online gestational age calculator easy to use. However, women completed the assessment using the electronic tablet provided by the study team rather than their own mobile phones. Although the website was optimised to be viewed on smaller screens, ease of use may therefore vary on devices with smaller screens. Most women thought having access to a self-assessing GA calculator would be helpful when considering having an abortion. When asked during the survey component of the study, one woman reported having an IUD in situ at the time of the study (which would be a contraindication to having a MA), but omitted to select the appropriate option when she initially completed the self-assessment. There are a number of possible explanations for this: either the research assistant phrased the question incorrectly; or the participant did not understand the question (she might have previously had an IUD in situ); or the research assistant accidentally selected the incorrect answer. Nevertheless, self-assessed questions need to be written in easily understood wording and might benefit from additional help topic information which could be selected if the person completing the assessment is unsure.

In low-resource settings and developing countries, last menstrual period (LMP) is often used to determine gestational age [21, 22]. A significant number of participants were able to recall their LMP date (71 %; 55/78). Although mean difference between self-calculated GA and ultrasound GA were not statistically significant by site, large variances in ability to accurately recall LMP were observed, particularly at the private NGO (see Table 2). These findings suggest that the use of LMP recall is useful in many but not all cases of GA estimation. Although all participants recruited from the public sector knew their GA, no differences were observed between sites and knowing GA did not translate into accurately recalling LMP. Furthermore, the low reported knowledge surrounding MA cut-off period legislation meant that most women would not have known the legal GA cut-off for having a MA. Although this study’s small sample size may not have adequate power to test validity, our emphasis was on the feasibility of women using an online gestational age calculator to self-assess their own GA. Self-calculated GA was overestimated within a margin of error (0.5 days) which is not clinically significant for MA.

Our study found a high self-reported rate of failed contraceptive use (41 %). In contrast, closer to 20 % of women with unintended pregnancies usually consider themselves to be using a form of contraception [30, 31]. It is possible that reported failed contraceptive methods might have been the result of incorrect use, for example forgetting to take an oral contraceptive pill, inconsistent condom use and missing appointment for injectable. However, reasons for failed contraceptive use were not explored as part of this study.

The high prevalence of feature/smart phones among participants adds strength to potential future mHealth interventions. Although not specifically from mobile phones, internet searches for abortion information prior to clinic visit were undertaken by 24 % of women. A recent study reported a high proportion of women who were initially denied an abortion at legal facilities went on to seek options for pregnancy termination outside of the legal system through internet searches – some of which could have led to unsafe abortion practices [9]. It therefore stands to reason that the ability to simultaneously accessing information and calculate GA online may well avoid delays, particularly for women nearing GA limit. Our study found very low levels of knowledge surrounding national abortion legislation in terms of cut-off periods for having an early MA. Providing women with readily accessible information might serve to increase knowledge surrounding abortion services and act as an enabler for women to access safe abortion services sooner.

### Limitations

The study sample size was relatively small, and all participants were recruited from health care facilities providing abortions. Study participants had therefore already decided to have an abortion and might have given their GA some consideration. Moreover, women who were recruited from the government facility already knew their GA prior to being recruited into the study. Although our study found that most women were able to recall their LMP, these findings might not be generalizable to other populations. The usability of the online gestational calculator was only tested on the electronic tablet provided by the study team and its use may therefore vary on devices with smaller screens.

## Conclusion

Timely access to information is an essential component of safe and effective abortion services. Advances in the availability of mobile technology and proliferation of mobile devices represent an unique opportunity to provide women with accurate and safe abortion information and services. Our study findings indicate that an online GA calculator would be both accurate and helpful for women considering having an abortion. Women could calculate their own GA based on LMP recall within 0.5 days when compared with confirmed GA by Ultrasound. An online GA calculator could potentially act as an enabler for women to access safe abortion services sooner.

## Abbreviations

CTOP: Choice on Termination of Pregnancy Act
GA: gestational age
IUD: intrauterine device
LMP: last menstrual period
MA: medical abortion
mHealth: mobile health technology
NGO: Non-Governmental Organisation
TOP: termination of pregnancy
WHO: World Health Organization
